# A Lightweight Identity Authentication Protocol for Vehicle Ad Hoc Network Based on PUF-Obfuscation

**DOI:** 10.3390/s26102971

**Published:** 2026-05-08

**Authors:** Jiaquan Song, Xiaofang Wang, Pengfei Lu

**Affiliations:** College of Information Science and Technology, Shihezi University, Shihezi 832003, China; songjiaquan@stu.shzu.edu.cn

**Keywords:** decentralized anonymous authentication, SDL PUF, CRP obfuscation, machine learning attack resilience, deep learning attack resilience, V2X communication

## Abstract

The rapid growth of Intelligent Transportation Systems (ITSs) necessitates secure and efficient Vehicle-to-Everything (V2X) communication. However, existing Physical Unclonable Function (PUF)-based schemes often suffer from modeling vulnerabilities and high overheads. This paper proposes a decentralized, dynamic, anonymous authentication protocol tailored for Vehicular Ad Hoc Networks (VANETs). By integrating Elliptic Curve Cryptography (ECC) with highly reliable Self-Adaption Deviation Locking PUFs (SDL PUFs), we design a dynamic Challenge–Response Pair (CRP) obfuscation mechanism. This mechanism effectively mitigates modeling threats, reducing the prediction success rate of machine learning (ML) and deep learning (DL) attacks by approximately 35% compared to raw SDL PUFs. The protocol ensures identity untraceability and forward secrecy through anonymous identifiers and ephemeral session keys. Security is formally verified under the Real-or-Random (ROR) model and validated using the AVISPA tool. Simulations in SUMO and Omnetpp demonstrate that the protocol is highly efficient, achieving a low computational overhead of 6.77 ms per entity and a communication cost of 192 bytes. Compared to state-of-the-art approaches, our solution provides superior robustness against advanced modeling attacks and significantly reduces latency, making it suitable for resource-constrained V2X environments.

## 1. Introduction

The profound integration of ITSs and autonomous driving technologies has established VANETs as the cornerstone of modern V2X communication [1]. In complex V2X interaction scenarios, network nodes exhibit significant performance heterogeneity: while autonomous vehicles are typically equipped with high-performance computing units capable of robust data processing, many Roadside Units (RSUs) and sensing nodes, such as smart traffic lights, remain resource-constrained due to deployment costs and power budgets [2,3]. This vast asymmetry in processing power renders traditional, heavyweight cryptographic suites impractical for high-stakes traffic environments where balancing low latency with lightweight execution is paramount.

In open wireless environments, identity authentication protocols serve as the primary defense in terms of securing communication [4]. Recently, the PUF has emerged as a promising hardware security primitive. By leveraging inherent random physical variations during silicon manufacturing to generate unique, unclonable “digital fingerprints”, PUFs provide an ideal security foundation for heterogeneous devices [5]. PUFs are generally classified into two types: Weak PUFs are primarily used for key generation and include the RO PUF [6], with uniqueness improved via Look-Up Table (LUT) self-comparison; lightweight RO PUFs [7] based on XOR gates; the area-efficient Loop PUF [8]; and the Transient Effect Ring Oscillator (TERO) PUF [9], which requires complex calibration. Conversely, strong PUFs are better suited for authentication, with examples including the 4:1 MUX APUF [10] architecture that leverages LUT6 primitives for high hardware utilization.

Despite these advancements, existing protocols [11] remain vulnerable to evolving adversarial tactics. Research indicates that storing CRPs in plaintext format within databases makes systems susceptible to physical capture attacks. More alarmingly, ML and DL attacks can now predict strong PUF responses with high precision. While the Dependency Chain mechanism (DC-PUF) [12] limits CNN modeling accuracy to approximately 58%, its reliability degrades significantly over successive authentication rounds. Furthermore, traditional schemes struggle with Ephemeral Secret Leakage (ESL) and quantum threats like Shor’s algorithm. For instance, while the ECC-based protocol [13] optimizes the total computational cost to 8.891 ms, it lacks targeted defense against modeling attacks. Similarly, the authors of [14] attempted to combine the PUF and ECC to enhance anonymity, but with 24.678 ms latency, the proposed approach remains redundant for dynamic V2X environments. Even the recently proposed EA2S2KA scheme [15] faces challenges in maintaining robustness under extreme environmental conditions.

To address these limitations, our paper proposes a decentralized anonymous authentication protocol based on the Self-adaption Deviation Locking PUF (SDL PUF) [16]. By utilizing the deviation locking mechanism of Self-Timed Rings (STRs), the SDL PUF achieves a zero bit-error rate across a wide temperature range of 0∼80 °C, providing exceptional environmental robustness [16]. The primary contributions of this work are summarized as follows:

- We propose a decentralized V2X authentication protocol leveraging the SDL PUF. By restricting the Trusted Authority (TA) to the registration phase, the architecture eliminates single-point bottlenecks, while the SDL PUF ensures a zero bit-error rate across wide temperature and voltage ranges.
- We design the DRO-Obfuscate algorithm, combining ECC and the SDL PUF to enable non-linear dynamic updates of Challenge–Response Pairs (CRPs). This synergism disrupts CRP logical correlation, reducing machine learning and deep learning prediction accuracy by approximately 35% compared to conventional schemes.
- We rigorously verify the protocol’s security under the ROR model and AVISPA tool, confirming its resilience against impersonation, replay, and ephemeral secret leakage. Performance evaluations demonstrate a low single-entity computational cost of 6.77 ms and communication overhead reductions of 23.85% and 72.57% against the models proposed in [13] and [14] respectively, achieving superior environmental robustness compared to the model proposed in [15].

## 2. Related Works

The quest for enhanced privacy in VANETs began with Public Key Infrastructure (PKI). In 2007, Raya et al. [17] proposed an aggregate signature scheme based on PKI to strengthen system anonymity, followed by Lu et al. [18] in 2008, who designed an anonymous PKI-based identity protection framework. However, these early paradigms were plagued by the prohibitive overhead of certificate management. To mitigate this, Zhang et al. [19] introduced an identity-based conditional privacy scheme. Seeking further performance optimization, Das et al. [20] explored a dual-factor authentication mechanism combining passwords and smart cards, yet Nyang et al. [21] later demonstrated its vulnerability to offline guessing attacks. Collectively, while these foundational works [17,18,19,20,21] established basic security requirements, their reliance on computationally intensive primitives—such as bilinear pairings or RSA—renders them ill-suited for resource-constrained On-Board Units (OBUs). Furthermore, they remain susceptible to single points of failure at the TA and physical exposure of stored cryptographic keys.

To address the inherent risks of physical key storage, PUFs have emerged as a promising hardware security primitive for VANETs. Guajardo et al. [22] pioneered the use of SRAM PUFs in FPGA environments for privacy protection, though their approach overlooks PUF reliability under environmental noise and lacks robustness against modeling attacks. In 2010, Sadeghi et al. [23] utilized bilinear pairings to protect PUF CRPs against ML modeling; however, this came at a staggering computational cost and failed to resolve the underlying reliability issues. In 2012, reliability was addressed by Van Herrewege et al. [24] through a Reverse Fuzzy Extractor, but this introduced susceptibility to replay and ML modeling attacks. Similarly, Rostami et al. [25] improved efficiency by discarding fuzzy extractors in favor of random response subsets, yet challenges regarding robustness and scalability persisted. In summary, these early PUF integrations [22,23,24,25] were hindered by the storage burden of massive CRP databases, susceptibility to noise, and a lack of ECC integration.

Subsequent research sought to overcome standalone PUF limitations by integrating ECC. In 2015, He et al. [26] optimized batch verification latency in ECC-based VANETs, though they did not focus on the intrinsic properties of the PUF itself. In 2016, Yu et al. [27] combined ECC with locking techniques to enhance ML resistance, yet once again bypassed the issue of PUF reliability. In 2019, while Gope et al. [28] avoided explicit CRP storage, their architecture remained vulnerable to insider threats. In the same vein, Yanambaka et al. [29] and Long et al. [30] relied on plaintext CRP transmissions, which are easily exploited by ML modeling. Although Chen et al. [31] improved security by encrypting CRPs via ECC, the resulting latency and the absence of a parameter update mechanism limited their model’s practical deployment. Furthermore, despite efficiency gains, these schemes [26,27,28,29,30,31] generally fail to defend against ESL attacks and remain tethered to a centralized TA for key generation.

To eliminate this TA dependency, Sutrala et al. [32] incorporated biometrics for mutual authentication, though at the cost of high design complexity. In 2022, Chaudhry et al. [33] developed a lightweight ECC protocol that, while resilient to common attacks, lacked dynamic parameter updates. In 2023, Xie et al. [34] and Wei et al. [35] leveraged smart contracts for TA-free Authentication and Key Agreement (AKA), albeit with heavy computational overhead. Liang et al. [36] proposed a TA-free PUF-ECC protocol, but the use of fixed pseudonyms introduced location-tracking risks. In 2024, Rostampour et al. [37] and Kumari et al. [38] proposed privacy-enhanced and lightweight authentication schemes for smart grids, but the proposed schemed do not address PUF reliability or resistance to modeling attacks in VANETs. While Reddy et al. [39] and Liu et al. (2025) [40] proposed decentralized protocols with partial physical resilience, the proposed approaches remain unable to safeguard sensitive parameters in the event of total key leakage. Thus, despite the shift toward decentralization [32,33,34,35,36,37,38,39,40], the trade-off between computational and communication overhead and the balance between privacy and traceability remain unresolved.

Recent literature has delved deeper into hardware-rooted security. Li et al. [41] deployed PUFs on both vehicles and RSUs to eliminate long-term key storage, while Men et al. [42] introduced real-time CRP generation. However, these approaches are hindered by TA involvement and a lack of scalability. In 2025, Li et al. [43] utilized SSL PUFs to improve reliability, but their scheme—along with those proposed in [41,42,43]—remains susceptible to ML modeling. Ponnuru et al. [44] achieved stronger protection by fusing blockchain, ECC, and PUFs, yet the time complexity remains a barrier for real-time V2X applications. Shang et al. [13] proposed an ECC protocol resilient to insider and ESL attacks, but the absence of PUF-based hardware security leaves it vulnerable to algorithm-specific ML modeling and quantum threats. Wang et al. [14] achieved a terminal latency of 2.45 ms through computation offloading; however, the massive communication overhead restricts the model’s use in bandwidth-sensitive scenarios. Finally, in 2026, Li et al. [15] presented the EA2S2KA scheme, which achieves optimal computational costs but does not consider the reliability of the PUF and its resistance to modeling attacks.

In conclusion, existing solutions have yet to achieve seamless integration of high-reliability PUFs, lightweight ECC, and robust resistance against both ML modeling attacks and various internal attacks, including ESL and insider threats. The emergence of the SDL PUF [16] provides a novel trajectory for the synergistic optimization of security, efficiency, and hardware-rooted trust, serving as the primary motivation for this research.

### Motivation

This paper enhances the PUF-based authentication protocols proposed by Men et al. [42], focusing on reducing computational and storage overhead while achieving several key security goals. These include mutual authentication to prevent impersonation attacks, session key establishment to ensure the confidentiality of communications, and forward and backward secrecy to protect past and future session keys from being exposed. The protocol is designed to resist modeling attacks leveraging ML/DL while ensuring anonymity and unlinkability to protect vehicle identities and driver privacy. Additionally, the scheme is robust against common attacks such as replay, impersonation, MITM, and DoS attacks [45], ensuring security in open vehicular networks. The protocol also emphasizes lightweight efficiency, making it suitable for resource-constrained OBUs [2] and RSUs [46], supporting large-scale deployment in VANETs.

## 3. PUF Secure Binding and Response Extraction

**Figure 1:**
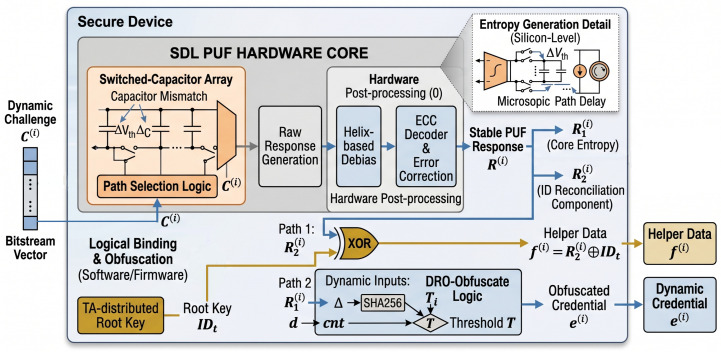
Hardware-Rooted Secure Binding and Response Extraction.

To establish a resilient hardware root of trust and mitigate modeling vulnerabilities [47,48], we implement an integrated response extraction and binding mechanism for the SDL PUF, as shown in Figure 1. Upon receiving a dynamic challenge ($C^{\left(i\right)}$), the SDL PUF hardware core exploits intrinsic silicon process variations in threshold voltage ($\Delta V_{th}$) and capacitance ($\Delta_{C}$) [16]. These microscopic physical differences manifest as unpredictable path-delay variations, which are digitized into a raw response bitstream. To guarantee environmental robustness and a near-zero bit-error rate, the hardware core performs tightly coupled post processing utilizing adaptive deviation locking and error correction [16]. The resulting stable PUF response ($R^{\left(i\right)}$) is uniquely bound to the device’s physical microstructure.

Immediately following extraction, the response is transformed within a secure logical boundary to eliminate modeling attack surfaces. $R^{\left(i\right)}$ is split into two functional components: $R_{1}^{\left(i\right)}$ (core entropy) and $R_{2}^{\left(i\right)}$ (ID reconciliation). Two concurrent security paths are then executed:

- Path 1: Root Key Reconciliation. The device reconstructs the TA-distributed root key ($ID_{t}$) by computing $f^{\left(i\right)}=R_{2}^{\left(i\right)}⊕ID_{t}$, where $f^{\left(i\right)}$ is stored helper data.
- Path 2: Dynamic Credential Obfuscation. $R_{1}^{\left(i\right)}$ serves as high-entropy input for the non-linear DRO-Obfuscate logic. This component is combined with an ephemeral scalar (*d*) and a dynamic offset (Δ) to derive the obfuscated credential ($e^{\left(i\right)}$).

This integrated mechanism ensures that the raw PUF fingerprint is never exposed in plaintext or persistent storage. The complete procedure effectively disrupts the logical correlation between challenges and responses to resist advanced ML/DL modeling attacks. The detailed steps are provided in Section 5.5.

## 4. System and Adversary Model

**Figure 2:**
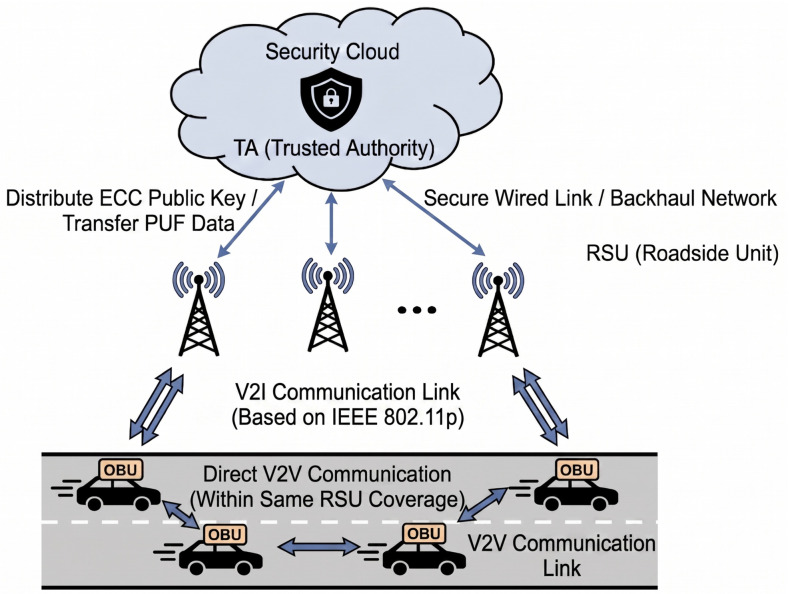
Architectural Architectural diagram of a V2X system based on IEEE 802.11p [1].

For our protocol, the considered system model is illustrated in Figure 2, consisting of three main entities: a TA, RSUs, and vehicles.

### 4.1. System Trust Assumptions

The system architecture incorporates three entities with logically segregated roles to facilitate hardware-anchored decentralized trust [49]. The TA acts as an offline root of trust, restricted to initial device registration and credential binding [50,51]. By design, the TA is strategically decoupled from real-time operations, participating in neither online authentication nor key agreement, which effectively eliminates single-point bottlenecks. During the online phase, RSUs and vehicles (OBUs) function as autonomous security nodes. Although considered semi-trusted due to potential storage exposure, both entities leverage integrated SDL PUFs to provide hardware-level tamper resistance. This enables distributed, mutual verification and pseudonym generation without reliance on continuous TA connectivity, ensuring identity protection and physical unclonability, even under compromise.

### 4.2. System Architecture

Unless stated otherwise, we adopt a Dolev–Yao-style [51] network adversary with extended device-access capabilities. Specifically, its capabilities include the following:

- **Single-end storage exposure:** The adversary can compromise and read the persistent or volatile storage of an RSU or a vehicle at any given time but cannot simultaneously compromise both entities.
- **Full-channel control:** The adversary can eavesdrop, capture, intercept, replay, delay, drop, inject, or modify any messages on V2X links.
- **Physical and modeling attacks:** The adversary may conduct side-channel or invasive physical attacks and utilize machine learning- and deep learning-based models to extract or infer parameters stored in vehicles and RSUs.

### 4.3. Security Goals

Based on the aforementioned system and threat models, the proposed protocol is required to achieve the following security objectives:

(1) **Mutual Authentication:** Both vehicles and RSUs must mutually verify each other’s [52] legitimacy to prevent impersonation attacks.

(2) **Session Key Freshness and Confidentiality:** Each authentication session must establish a unique and secret session key, ensuring the confidentiality of subsequent V2X communications.

(3) **Forward and Backward Secrecy:** The compromise of a long-term secret must not endanger past session keys [53], and leakage of a session key must not affect future sessions.

(4) **Resistance to Modeling Attacks:** The protocol must ensure robustness against ML/DL-based modeling attempts on PUF CRPs [54], preserving the unpredictability of responses.

(5) **Anonymity and Unlinkability:** Vehicles must remain anonymous during authentication, and adversaries should not be able to link multiple sessions to trace a specific vehicle’s identity or movement.

(6) **Integrity and Anti-Replay Protection:** The protocol should detect and prevent replayed, modified, or injected messages, thereby defending against replay and MITM attacks [55].

(7) **DoS Resistance and Lightweight Efficiency:** The design must minimize computational and communication overhead, enabling efficient execution on resource-constrained OBUs and RSUs while reducing susceptibility to DoS attacks.

## 5. The Proposed Protocol Suite

Table 1 lists the notation used in the protocol. This work uses random numbers and hash functions, adds a challenge/response mechanism to RSUs and vehicles, and develops a TA-free authentication and key agreement protocol for the Internet of V2X [56]. The protocol uses identity-based credentials and dynamic challenges to implement five core phases.

### 5.1. Vehicle Registration Phase

As shown in Table 2, each vehicle ($V_{i}$) registers with the TA before joining the system. A secure channel is assumed between $V_{i}$ and the TA during registration. Let *P* be the base point of an elliptic curve group of prime order *n*.

(1) **Vehicle → TA:** $V_{i}$ chooses an SDL PUF challenge ($C_{v}$) and sends its identifier ($ID_{i}$) and $C_{v}$ to the TA over a secure channel.

(2) **TA processing and response:** Upon receiving $\left(ID_{i},C_{v}\right)$, the TA verifies $ID_{i}$, samples a random nonce ($\alpha_{i}\in Z_{n}^{*}$), selects a TA-specific challenge ($C_{T}$), derives a TA of the SDL PUF response key ($ID_{t}={\mathrm{PUF}}_{\mathrm{TA}}\left(C_{T}\right)$), and computes the public token ($X_{i}=\alpha_{i}P$). The TA returns $\left(\alpha_{i},ID_{t},X_{i}\right)$ to $V_{i}$ via the secure channel.

(3) **Vehicle sealing of helper data:** $V_{i}$ evaluates its SDL PUF to obtain $R_{v}:={\mathrm{PUF}}_{V}\left(C_{v}\right)$ and splits it as $R_{v}=R_{v1}\|R_{v2}$. It then computes $e_{i}=R_{v1}⊕\alpha_{i}$,$f_{i}=R_{v2}⊕ID_{t}$.

Finally, $V_{i}$ securely stores $\left\{e_{i},C_{v},f_{i}\right\}$ as local helper data and publishes $X_{i}$ on a public directory for later lookup.

### 5.2. RSU Node Registration Phase

As shown in Table 3, RSUs are required to register with the TA before joining the vehicular network. The registration process is performed over a secure channel and runs concurrently with vehicle registration. The detailed procedure is outlined as follows:

(1) **RSU → TA:** The RSU $ID_{j}$ selects an SDL PUF challenge ($C_{RSU}$) and attaches its identity ($ID_{j}$). The RSU then transmits $\left(ID_{j},C_{RSU}\right)$ to the TA through a secure channel.

(2) **TA processing and response:** Upon receiving $ID_{j}$ and $C_{RSU}$, the TA verifies $ID_{j}$, randomly selects $\beta_{j}\in Z_{n}^{*}$, chooses the same challenge value ($C_{T}$), and computes a TA-specific response ($ID_{t}={\mathrm{PUF}}_{TA}\left(C_{T}\right)$). It then computes the public token as $Y_{j}=\beta_{j}P$. Finally, the TA returns $\left(\beta_{j},ID_{t},Y_{j}\right)$ to the RSU via the secure channel.

(3) **RSU sealing of helper data:** The RSU computes $R_{RSU}={\mathrm{PUF}}_{\mathrm{RSU}}\left(C_{RSU}\right)$ and splits it into two parts ($R_{RSU}=R_{RSU1}\|R_{RSU2}$). It then computes $e_{j}=R_{RSU1}⊕\beta_{j}$, $f_{j}=R_{RSU2}⊕ID_{t}$. The RSU securely stores $\left\{e_{j},C_{RSU},f_{j}\right\}$ as helper data and publishes $Y_{j}$ over the public channel.

### 5.3. Mutual Authentication and Key Agreement Phase

As shown in Table 4, during this phase, the RSU and the vehicle perform mutual authentication and establish a session key without the involvement of the TA [57]. The detailed process is outlined as follows:

(1) **Vehicle → RSU:** The vehicle generates random numbers ($d_{i},\gamma_{i},r_{i}\in Z_{q}^{*}$), selects an SDL PUF challenge ($C_{V}$), and computes the response ($R_{V}={\mathrm{PUF}}_{V}\left(C_{V}\right)$). It then derives $\alpha_{i}=e_{i}⊕R_{V1}$ and ${\mathrm{ID}}_{t}=f_{i}⊕R_{V2}$. The vehicle computes the PUF-bound temporary private key ($d_{i}^{\prime }=H\left(d_{i}∥R_{V}\right)$) and the corresponding public key ($D_{i}=d_{i}^{\prime }\cdot P$). It also generates a pseudonym (${\mathrm{PID}}_{i}={\mathrm{ID}}_{i}⊕\gamma_{i}$) and a timestamp ($T_{i}$). The authentication value is calculated as $A_{i}=H({\mathrm{PID}}_{i}∥{\mathrm{ID}}_{i}∥{\mathrm{ID}}_{j}∥\alpha_{i}Y_{j}∥D_{i}∥T_{i}∥r_{i})$. Finally, the vehicle transmits ${\mathrm{MSG}}_{1}=\left\{{\mathrm{PID}}_{i},A_{i},D_{i},r_{i},T_{i}\right\}$ to the RSU via the public channel.

(2) **RSU → Vehicle:** Upon receiving ${\mathrm{MSG}}_{1}$, the RSU checks the freshness of $T_{i}$ and verifies ${\mathrm{ID}}_{j}$. It then generates random numbers ($d_{j},\gamma_{j},r_{j}\in Z_{q}^{*}$), selects its own SDL PUF challenge ($C_{\mathrm{RSU}}$), and computes $R_{\mathrm{RSU}}={\mathrm{PUF}}_{\mathrm{RSU}}\left(C_{\mathrm{RSU}}\right)$. The RSU derives $\beta_{j}=R_{\mathrm{RSU}1}⊕e_{j}$ and ${\mathrm{ID}}_{t}=R_{\mathrm{RSU}2}⊕f_{j}$. It computes the PUF-bound temporary private key ($d_{j}^{\prime }=H\left(d_{j}∥R_{\mathrm{RSU}}\right)$) and the corresponding public key ($D_{j}=d_{j}^{\prime }\cdot P$), as well as the Diffie–Hellman shared secret ($K_{DH}=d_{j}^{\prime }\cdot D_{i}$). The RSU then recomputes the authentication value as $A_{j}=H({\mathrm{PID}}_{i}∥{\mathrm{ID}}_{i}∥{\mathrm{ID}}_{j}∥X_{i}\beta_{j}∥D_{i}∥T_{i}∥r_{i})$ and checks whether $A_{j}$ equals the received $A_{i}$. If the verification fails, the protocol is aborted. Otherwise, the RSU generates a new timestamp ($T_{j}$), computes ${\mathrm{TID}}_{t}={\mathrm{ID}}_{t}⊕\left(D_{i}\cdot d_{j}^{\prime }\right)$, updates the pseudonym (${\mathrm{PID}}_{i}^{1}={\mathrm{PID}}_{i}⊕\gamma_{j}$), and calculates $\rho_{j}=H({\mathrm{PID}}_{i}^{1}∥{\mathrm{TID}}_{t}∥A_{j}∥T_{i}∥T_{j}∥r_{j})⊕\left(D_{i}\cdot d_{j}^{\prime }\right)$. It sends ${\mathrm{MSG}}_{2}=\left\{{\mathrm{PID}}_{i}^{1},\rho_{j},D_{j},r_{j},T_{j}\right\}$ back to the vehicle.

(3) **Vehicle → RSU:** The vehicle verifies the freshness of $T_{j}$ and computes the Diffie–Hellman shared secret ($K_{DH}=d_{i}^{\prime }\cdot D_{j}$). It then derives ${\mathrm{TID}}_{t}={\mathrm{ID}}_{t}⊕\left(D_{j}\cdot d_{i}^{\prime }\right)$ and computes the expected value, i.e., $\rho_{i}=H({\mathrm{PID}}_{i}^{1}∥{\mathrm{TID}}_{t}∥A_{i}∥T_{i}∥T_{j}∥r_{j})⊕\left(D_{j}\cdot d_{i}^{\prime }\right)$. If $\rho_{i}$ matches the received $\rho_{j}$, the vehicle proceeds to generate a fresh timestamp ($T_{i}^{*}$) and derives the session key ($K=H(K_{DH}∥{\mathrm{PID}}_{i}∥{\mathrm{PID}}_{i}^{1}∥T_{i}∥T_{j}∥r_{i}∥r_{j})$). It then computes the key confirmation token ($\tau_{i}=H\left(K∥T_{i}^{*}\right)$). Additionally, the vehicle selects a new SDL PUF challenge ($C_{V}^{\prime }$), computes $R_{V}^{\prime }={\mathrm{PUF}}_{V}\left(C_{V}^{\prime }\right)$, and updates its helper data $\left(f_{i},e_{i}\right)$ accordingly. Finally, it sends ${\mathrm{MSG}}_{3}=\left\{\tau_{i},T_{i}^{*}\right\}$ to the RSU.

(4) **RSU Confirmation:** The RSU checks the freshness of $T_{i}^{*}$ and computes the same session key ($K=H(K_{DH}∥{\mathrm{PID}}_{i}∥{\mathrm{PID}}_{i}^{1}∥T_{i}∥T_{j}∥r_{i}∥r_{j})$). It then calculates $\tau_{j}=H\left(K∥T_{i}^{*}\right)$ and verifies that $\tau_{j}$ equals the received $\tau_{i}$. If the verification succeeds, the RSU also updates its helper data $\left(f_{j},e_{j}\right)$ using a new SDL PUF challenge ($C_{\mathrm{RSU}}^{\prime }$) and response ($R_{\mathrm{RSU}}^{\prime }$). At this point, mutual authentication and session key agreement are successfully completed, and both parties share the same session key (*K*).

### 5.4. Parameter Update Phase

After the RSU and the vehicle complete one round of mutual authentication and key agreement, both parties refresh their local parameters to ensure long-term security and session independence.

(1) **Vehicle-side update:** The vehicle replaces the previous round’s long-term secret ($\alpha_{i}$) with the newly generated random value ($d_{i}$), updates the TA-derived secret (${\mathrm{ID}}_{t}$) to the fresh token (${\mathrm{TID}}_{t}$) obtained during the session, and re-seals its helper data using the new SDL PUF challenge–response pair ($\left(C_{V}^{\prime },R_{V}^{\prime }\right)$): $f_{i}=R_{V2}⊕{\mathrm{TID}}_{t}$, $e_{i}=R_{V1}⊕d_{i}$. The tuple expressed as $\left\{e_{i},C_{V}^{\prime },f_{i}\right\}$ overwrites the old helper data, and the public token ($X_{i}$) is replaced by $D_{i}$ (the temporary public key from the session).

(2) **RSU-side update:** Similarly, the RSU replaces its previous long-term secret ($\beta_{j}$) with the newly generated random value ($d_{j}$), updates ${\mathrm{ID}}_{t}$ to ${\mathrm{TID}}_{t}$, and re-computes its helper data using a new SDL PUF challenge–response pair $\left(C_{\mathrm{RSU}}^{\prime },R_{\mathrm{RSU}}^{\prime }\right)$: ($f_{j}=R_{\mathrm{RSU}2}⊕{\mathrm{TID}}_{t}$, $e_{j}=R_{\mathrm{RSU}1}⊕d_{j}$). The tuple expressed as $\left\{e_{j},C_{\mathrm{RSU}}^{\prime },f_{j}\right\}$ overwrites the old helper data, and the public token ($Y_{j}$) is replaced by $D_{j}$.

(3) **Public tokens:** As part of the previous mutual authentication and key agreement round, the public tokens are rotated: $X_{i}$ and $Y_{j}$ are replaced by $D_{i}$ and $D_{j}$ respectively. This periodic refresh prevents linkage across sessions and strengthens forward/backward secrecy.

### 5.5. SDL PUF Obfuscation of the User

In the registration and authentication process of the vehicle and RSU, when the number of vehicles and RSUs exceeds a threshold (*T*), the system becomes vulnerable to ML and DL attacks. To defend against such attacks, we employ Algorithm 1 (DRO-Obfuscate) to update the $e_{i}$ and $e_{j}$ parameters.
**Algorithm 1** DRO-Obfuscate $\left(C,d,T_{i},\Delta ,cnt,T\right)$1:   cnt←02:   cnt←cnt+13:   $R_{1}\leftarrow \mathrm{first}16\mathrm{Bytes}\;\mathrm{SDL}\;\mathrm{PUF}\left(C\right)$4:   while cnt>T do5:        $tmp\leftarrow \Delta ∥T_{i}∥d$6:        Δ←SHA256(tmp)[0..15]7:        $e\leftarrow R_{1}⊕d⊕\Delta$8:        break9:   end while10:   while cnt≤T do11:        $e\leftarrow R_{1}⊕d$12:        break13:   end while14:   return (e,Δ,cnt)

In Algorithm 1, *d* takes a random value from the current round of the protocol process, while $\left(C,R_{1}\right)$ refers to the challenge–response pair generated by each party’s SDL PUF. The value of $T_{i}$ is the timestamp of the current session, and both the vehicle and RSU store a dynamic offset (Δ), which is updated during each session.

## 6. Formal Security Analysis

To ensure the security of the protocol, this section provides the formal security analysis and proof of the V2X protocol proposed in Section 5 under the ROR model. We derive step-by-step queries to formally prove that the protocol satisfies the required session key secrecy and mutual authentication security properties. Under clearly defined security assumptions, this section demonstrates how the advantage of an adversary (A) is gradually reduced through each game transition.

**Players:** In the tripartite V2X environment, we define the protocol (P) as consisting of three types of entities: TA, RSU, and *V*. During protocol execution, the Trusted Authority, Roadside Unit, and Vehicle are instantiated as $T_{k}$, $RSU_{j}$, and $V_{i}$, respectively. Let $\Pi_{V_{i}}^{l}$ and $\Pi_{RSU_{j}}^{m}$ denote the *l*-th and *m*-th instances (oracles) of vehicle $V_{i}$ and $RSU_{j}$, respectively. These instances serve as the logical execution units of the protocol.

**Queries:** These query statements aim to simulate the capabilities of a real A, with the following query types available to A [13]:

1. $\mathrm{Execute}\;(V_{i},{\mathrm{RSU}}_{j})$: This query simulates passive eavesdropping. A can obtain all messages $\left\{{\mathrm{MSG}}_{1},{\mathrm{MSG}}_{2},{\mathrm{MSG}}_{3}\right\}$ honestly exchanged between the two parties.
2. Send(P,Π,m): This query simulates active attacks. A masquerades as a peer (*P*) of instance Π, sends a forged or modified message *m* to instance Π, and obtains the response.
3. Reveal(Π): This query simulates session key leakage. If Π has accepted, A obtains the current session key (*K*).
4. PUF(dev,C): This query simulates the computation of a physical unclonable function. A provides a challenge (*C*) and obtains the simulated response (*R*).
5. $\mathrm{Corrupt}\;(P_{i})$: This query simulates the ability of A to compromise the internal storage or secrets of an entity, including the following two scenarios:
  - For $P_{i}=V_{i}$, A can obtain auxiliary data $\left\{e_{i},C_{V},f_{i}\right\}$ stored in the OBU.
  - For $P_{i}={\mathrm{RSU}}_{j}$, A can obtain the RSU’s local secrets $\left\{e_{j},C_{\mathrm{RSU}},f_{j}\right\}$.
6. Test(Π): This query is used to define the semantic security of the session key rather than to simulate the adversary (A). It is executed only once on a fresh session. If instance Π lacks a session key or the session is not fresh, it returns ⊥. Otherwise, a random bit (*b*) is chosen. If b=1, the real key (*K*) is returned; if b=0, a random string of equal length is returned.

In addition, it is necessary to define Partnering, Freshness, Semantic Security, and the Computational Difficulty Problem [13].

**Partnering:** Two instances ($\Pi_{V_{i}}^{l}$ and $\Pi_{RSU_{j}}^{m}$) are said to be in a partnering state if and only if (1) both entities have successfully completed mutual identity confirmation; (2) both entities share the same session identifier, i.e., $SID=(MSG_{1}||MSG_{2}||MSG_{3})$; and (3) the partner identifier (pid) of $\Pi_{V_{i}}^{l}$ is $\Pi_{RSU_{j}}^{m}$ and the pid of $\Pi_{RSU_{j}}^{m}$ is $\Pi_{V_{i}}^{l}$.

**Freshness:** An instance (Π) is considered fresh if its session key has not been revealed, and A does not simultaneously compromise the long-term secrets of both communicating parties. Specifically, (1) identity confirmation is passed, and the session key is not leaked; (2) A has not executed a Reveal query against Π or its partner; (3) the $Corrupt(V_{i})$ query has been executed, at most, once; and (4) A has not simultaneously compromised the long-term secrets of both communicating parties.

**Semantic Security:** The security of the session key (SK) is defined by this concept. During the execution of protocol P, A can perform a polynomial number of Execute, Send, and Reveal queries and a single Test query on a fresh instance. At the end of the game, A must guess the bit (*b*). A correct guess means A successfully broke the semantic security of the protocol, denoted as Pr[Succ(A)]. The advantage of A in breaking the semantic security is calculated as

(1) $$Adv_{A}^{P}=\left|2\mathrm{Pr}\left[Succ\right]-1\right|\leq \epsilon .$$

**Elliptic Curve Discrete Logarithm Problem (ECDLP):** In an elliptic curve (*E*) defined over a finite field ($F_{p}$) [47], given a base point (*P*; of prime order (*n*)) and a public key point (*Q*), for any PPT adversary (A), it is computationally infeasible to solve for the scalar (*d*), given *P* and *Q*. Q=d·P, where $d\in Z_{n}^{*}$ and $P,Q\in E(F_{p})$ [48]. The advantage of A in solving *d* is defined as [13]

(2) $$Adv^{\mathrm{ECCDLP}}\left(A\right)=\mathrm{Pr}\left[A\left(P,dP\right)=d\right]<\epsilon .$$

**Elliptic Curve Computational Diffie–Hellman Problem (ECCDHP):** Given three points {P,aP,bP} on an elliptic curve, where *a* and *b* are unknown random scalars, for a PPT adversary (A), computing the shared secret point (abP) is extremely difficult [47]. In the protocol proof, this ensures that even if an attacker intercepts the ephemeral public keys of both parties, they cannot derive the shared key without the private keys [13]. Given, P,A=a·P,B=b·P, solve C=a·b·P=a·B=b·A. The advantage of A is defined as

(3) $$Adv^{\mathrm{ECCDHP}}\left(A\right)=\mathrm{Pr}\left[A\left(P,aP,bP\right)=abP\right]<\epsilon .$$

**Theorem 1.** 
*Let A be a PPT adversary running in polynomial time, performing, at most, $q_{s}$ Send queries, $q_{e}$ Execute queries, and $q_{h}$ Hash queries against protocol P. Let $Adv^{\mathrm{ECCDHP}}\left(A\right)$ and $Adv^{\mathrm{ECCDLP}}\left(A\right)$ denote the advantages of A in breaking the ECCDHP and ECDLP problems, respectively. Let l be the bit length of the hash function, n be the order of the elliptic curve group, and negl(λ) be a negligible function representing the modeling unpredictability of the SDL PUF. Under the ROR model, the advantage of A in breaking the session key security of the protocol satisfies the following:*

(4) $$\begin{array}{cc} Adv_{A}^{P}\leq & \frac{q_{h}^{2}}{2^{l}}+\frac{{\left(q_{s}+q_{e}\right)}^{2}}{n}+2q_{s}\left(Adv_{A}^{\mathrm{PUF}}+\mathrm{negl}\left(\lambda \right)\right)\\ \; & +2\cdot \max\{Adv^{\mathrm{ECCDHP}}\left(A\right),Adv^{\mathrm{ECCDLP}}\left(A\right)\} \end{array}$$

**Proof.**  To rigorously prove the semantic security of the proposed protocol, we define a sequence of games ($G_{i}\left(i=0,1,\ldots ,5\right)$) and let $S_{i}$ be the event in which A successfully guesses the bit (*b*) in the Test query for $G_{i}$.

**Game $G_{0}$**: This game corresponds to the real attack by A against our protocol in the random oracle model. The challenger simulates all oracles (Execute,Send,Reveal,Corrupt,Test) honestly. By definition, we have

(5) $$Adv_{A}^{P}=\left|2\mathrm{Pr}\left[S_{0}\right]-1\right|.$$

**Game $G_{1}$**: This game models a passive eavesdropping attack. A intercepts the communication transcripts $\left\{MSG_{1},MSG_{2},MSG_{3}\right\}$ via the $\mathrm{Execute}(V_{i},RSU_{j})$ query. To compute the session key ($K=H(K_{DH}\|PID_{i}\|PID_{i}^{1}\|T_{i}\|T_{j}\|r_{i}\|r_{j})$), A must derive $K_{DH}=d_{i}^{\prime }\cdot d_{j}^{\prime }\cdot P$ from the public keys ($D_{i}$ and $D_{j}$). However, this is computationally infeasible due to the ECCDHP assumption. Thus, the success probability remains unchanged:

(6) $$\mathrm{Pr}\left[S_{1}\right]=\mathrm{Pr}\left[S_{0}\right]$$

**Game $G_{2}$**: This game simulates an active attack where A uses Send and Hash queries to induce collisions or forge messages. (1) HashCollisions: According to the birthday paradox, the probability of a collision in H(·) is, at most, $q_{h}^{2}/2^{l+1}$. (2) Nonce/Timestamp Collisions: The use of fresh nonces $\left\{d_{i},r_{i},d_{j},r_{j}\right\}$ and timestamps $\left\{T_{i},T_{j}\right\}$ ensures uniqueness, with collision probability bounded by ${\left(q_{s}+q_{e}\right)}^{2}/2n$. Applying the difference lemma, we obtain

(7) $$|\mathrm{Pr}\left[S_{2}\right]-\mathrm{Pr}\left[S_{1}\right]|\;\leq \frac{q_{h}^{2}}{2^{l+1}}+\frac{{\left(q_{s}+q_{e}\right)}^{2}}{2n}.$$

**Game $G_{3}$**: This game simulates the physical compromise and modeling attack. A executes $\mathrm{Corrupt}(V_{i})$ to extract $\left\{e_{i},C_{V},f_{i}\right\}$ from OBU storage. (1) SDL PUF Resilience: To derive the private key ($d_{i}^{\prime }=H\left(d_{i}\|R_{V}\right)$), A needs the PUF response ($R_{V}$). Since $e_{i}=R_{V1}⊕\alpha_{i}$, the extracted $e_{i}$ is logically blinded and provides no information about $R_{V}$. (2) DRO−Obfuscate Mechanism: Our dynamic obfuscation mechanism ensures that the logical correlation between challenges and responses is disrupted, bounding A’s ability to model the SDL PUF to a negligible function (negl(λ)). Thus, the success probability difference is

(8) $$|\mathrm{Pr}\left[S_{3}\right]-\mathrm{Pr}\left[S_{2}\right]|\;\leq q_{s}\cdot \left(Adv_{A}^{\mathrm{PUF}}+\mathrm{negl}\left(\lambda \right)\right).$$

**Game $G_{4}$**: In this game, the real shared secret ($K_{DH}=d_{i}^{\prime }\cdot d_{j}^{\prime }\cdot P$) is replaced by a truly random value (Z∈G). According to the protocol, the session key is derived as $K=H(K_{DH}\|PID_{i}\|PID_{i}^{1}\|T_{i}\|T_{j}\|r_{i}\|r_{j})$. The ability of A to distinguish $G_{4}$ from $G_{3}$ implies that A can solve the ECCDHP instance, given the public values of $D_{i}=d_{i}\cdot P$ and $D_{j}=d_{j}\cdot P$ from $MSG_{1}$ and $MSG_{2}$. Consequently, the probability of A distinguishing these two games is bounded by the advantage of solving computational hardness problems.

(9) $$|\mathrm{Pr}\left[S_{4}\right]-\mathrm{Pr}\left[S_{3}\right]|\;\leq \max\left\{Adv^{\mathrm{ECCDHP}}\left(A\right),Adv^{\mathrm{ECDLP}}\left(A\right)\right\}$$

**Game $G_{5}$**: Since the shared secret ($K_{DH}$) has been replaced by a truly random value (*Z*) in $G_{4}$, the session key (*K*) in this game is produced as K=H(Z‖…), which is now perfectly independent of any communication transcripts A has gathered. Thus, the probability of A successfully guessing the bit (*b*) in the Test query is

(10) $$\mathrm{Pr}\left[S_{5}\right]=\mathrm{Pr}\left[S_{4}\right]=\frac{1}{2}.$$

Based on the Equations (5) and (10), the advantage of A can be expressed as

(11) $$\frac{1}{2}Adv_{A}^{P}=|\mathrm{Pr}\left[S_{0}\right]-\frac{1}{2}|=|\mathrm{Pr}\left[S_{0}\right]-\mathrm{Pr}\left[S_{4}\right]|.$$

Employing the triangular inequality to decompose the differences between successive games yields

(12) $$\begin{array}{cc} |\mathrm{Pr}\left[S_{0}\right]-\mathrm{Pr}\left[S_{4}\right]|\;\leq & |\mathrm{Pr}\left[S_{0}\right]-\mathrm{Pr}\left[S_{1}\right]|+|\mathrm{Pr}\left[S_{1}\right]-\mathrm{Pr}\left[S_{2}\right]|\\ \; & +|\mathrm{Pr}\left[S_{2}\right]-\mathrm{Pr}\left[S_{3}\right]|+|\mathrm{Pr}\left[S_{3}\right]-\mathrm{Pr}\left[S_{4}\right]|. \end{array}$$

According to Equations (7)–(9), we obtain the final bound:

(13) $$\begin{array}{cc} Adv_{A}^{P}\leq & \frac{q_{h}^{2}}{2^{l}}+\frac{{\left(q_{s}+q_{e}\right)}^{2}}{n}+2q_{s}\left(Adv_{A}^{\mathrm{PUF}}+\mathrm{negl}\left(\lambda \right)\right)\\ \; & +2\cdot \max\{Adv^{\mathrm{ECCDHP}}\left(A\right),Adv^{\mathrm{ECDLP}}\left(A\right)\} \end{array}$$

Since all terms on the right side are negligible, the proposed protocol is formally proven to be secure under the ROR model. □

**Theorem 2** 
(Session Key Secrecy)**.** *Under the ROR model, let P be the proposed protocol and A be a PPT adversary that performs, at most, $q_{h}$ hash queries, $q_{s}$ Send queries, and $q_{e}$ Execute queries. Assuming the hash function (H(·)) is modeled as a random oracle and the underlying SDL PUF satisfies the physical unpredictability assumption, the advantage of A in breaking the session key secrecy of the protocol satisfies*

(14) $$\begin{array}{cc} Adv_{A}^{P}\leq & \frac{q_{h}^{2}}{2^{l}}+\frac{{\left(q_{s}+q_{e}\right)}^{2}}{n}+2q_{s}\left(Adv_{A}^{\mathrm{PUF}}+\mathrm{negl}\left(\lambda \right)\right)\\ \; & +2\cdot \max\{Adv^{\mathrm{ECCDHP}}\left(A\right),Adv^{\mathrm{ECCDLP}}\left(A\right)\}, \end{array}$$

*where l is the bit length of the hash output, n is the order of the elliptic curve group, and negl(λ) represents the negligible modeling advantage against the DRO-Obfuscate mechanism.*

**Proof.**  The proof is established through a sequence of games ($G_{0}$ to $G_{5}$). We start from the real attack in $G_{0}$ and gradually transition to $G_{5}$, where the session key (*K*) is replaced by a truly random string. Specifically, the gap between $G_{1}$ and $G_{2}$ accounts for the hash and nonce collision probabilities ($\left(\frac{q_{h}^{2}}{2^{l+1}}+\frac{{\left(q_{s}+q_{e}\right)}^{2}}{2n}\right)$). The transition from $G_{2}$ to $G_{3}$ incorporates the physical security of the OBU and the anti-modeling capability of the SDL PUF enhanced by the DRO-Obfuscate mechanism (Algorithm 1), contributing $q_{s}\left(Adv_{A}^{\mathrm{PUF}}+\mathrm{negl}\left(\lambda \right)\right)$. Finally, the gap between $G_{3}$ and $G_{5}$ (via $G_{4}$) is bounded by the computational hardness of the ECCDHP and ECDLP problems. By applying the triangular inequality, ($\frac{1}{2}Adv_{A}^{P}=\left|\mathrm{Pr}\left[S_{0}\right]-\mathrm{Pr}\left[S_{5}\right]\right|$), we derive the final advantage bound. Since all terms are negligible, the session key (*K*) is computationally indistinguishable from a random string. □

**Theorem 3** 
(Mutual Authentication)**.** *Under the random oracle model and the Dolev–Yao threat model, the proposed protocol ensures mutual authentication between vehicle $V_{i}$ and roadside unit $RSU_{j}$. Unless an attacker can solve the ECCDHP problem or forge SDL PUF outputs with non-negligible probability, any adversary (A) attempting to masquerade as a legitimate entity will fail the verification of authentication tags ($\left\{A_{i},\rho_{j},\tau_{i}\right\}$). The probability of a successful impersonation attack (Pr[Auth]) satisfies*

(15) $$Pr\left[\mathrm{Auth}\right]\leq \frac{q_{h}^{2}}{2^{l}}+\frac{{\left(q_{s}+q_{e}\right)}^{2}}{n}+2q_{s}\left(Adv_{A}^{\mathrm{PUF}}+\mathrm{negl}\left(\lambda \right)\right)+2\cdot Adv^{\mathrm{ECCDHP}}\left(A\right).$$

**Proof.**  The mutual authentication is guaranteed by the unforgeability of the authentication tags in the three-way handshake. (1) $V_{i}$ to $RSU_{j}$: The tag ($A_{i}$) involves the long-term secret term ($\alpha_{i}Y_{j}=\alpha_{i}\beta_{j}P$). Computing this term without the private key ($\alpha_{i}$, stored in PUF-protected memory) is equivalent to solving the ECCDHP problem. (2) $RSU_{j}$ to $V_{i}$: The tag ($\rho_{j}$) is masked by $D_{i}\cdot d_{j}^{\prime }$ and includes secret $ID_{t}$. An attacker cannot forge $\rho_{j}$ without the ephemeral private key ($d_{j}^{\prime }$) or the TA-distributed secret ($ID_{t}$), which is obfuscated by the PUF auxiliary data ($f_{i}=R_{V2}⊕ID_{t}$). (3) Key Confirmation: The tag expressed as $\tau_{i}=H\left(K\|T_{i}^{*}\right)$ in $MSG_{3}$ provides explicit confirmation that both parties have computed the same session key (*K*). As demonstrated in the game-based proof, the probability of A forging these tags is bounded by the hash collision probability, the PUF modeling advantage, and the ECCDHP hardness. Combined with the freshness check ($|T^{\prime }-T|<\Delta T$) which prevents replay attacks, the protocol ensures robust mutual authentication. □

### 6.1. Formal Security Analysis Using AVISPA Tool

**Figure 3:**
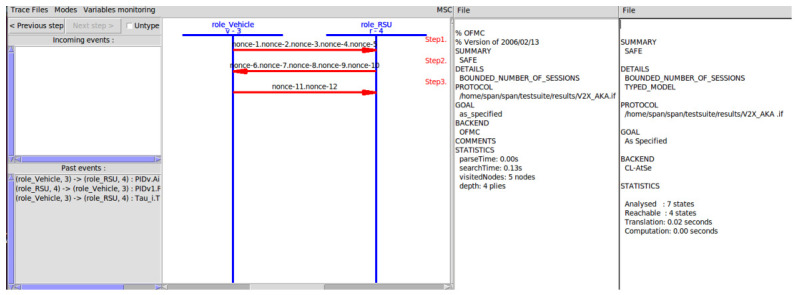
Simulation results from OFMC and CL-ATSE backend of AVISPA.

As illustrated in Figure 3, we utilize the Automated Validation of Internet Security Protocols and Applications (AVISPA) tool [57,58] to formally verify the proposed protocol’s session key secrecy and mutual authentication. Under the standard Dolev–Yao adversary model [59], the OFMC backend evaluated the protocol with a search depth of four plies and five visited nodes, while the CL-AtSe backend reported seven analyzed states and four reachable states [60]. Specifically, the four reachable states align precisely with the honest three-pass execution trace, whereas the additional analyzed states confirm that all active adversarial attempts (e.g., message injection or forgery) were successfully pruned by the protocol’s strict cryptographic bindings. Ultimately, both backends identically yield a “SAFE” summary, rigorously proving that the protocol is highly resilient against active network interventions and replay attacks [58].

### 6.2. Informal Security Analysis

This section evaluates the protocol’s heuristic resilience against common V2X security threats based on the interaction logic defined in Section 5.

(1) Resilience to Replay Attacks: The protocol incorporates fresh timestamps ($T_{i},T_{j},T_{i}^{*}$) and random nonces ($d_{i},d_{j},r_{i},r_{j}$) in every message. Any intercepted message from previous sessions will fail the freshness check ($|T^{\prime }-T|<\Delta T$), ensuring protection against replay.

(2) Mitigation of Impersonation Attacks: Legitimate identities are cryptographically bound to hardware secrets via tags $A_{i}$ and $\rho_{j}$. Without access to the internal SDL PUF response ($R_{V}$) or the TA-distributed secret ($ID_{t}$), an adversary cannot forge valid authentication tokens to masquerade as a vehicle or RSU.

(3) Protection Against MITM Attacks: All critical session parameters, including identities and nonces, are integrity-protected through hash-based bindings in $MSG_{1}$ and $MSG_{2}$. Any unauthorized modification of the messages in transit will result in a verification failure.

(4) Mutual Authentication and Key Agreement: The protocol achieves mutual trust through a three-pass handshake. The final verification of key confirmation tokens ($\tau_{i},\tau_{j}$) ensures that both entities have computed an identical and fresh session key (*K*).

(5) Guarantee of User Anonymity: To prevent tracking, vehicles utilize dynamic pseudonyms ($PID_{i},PID_{i}^{1}$) updated in every session. The link between pseudonyms and the real $ID_{i}$ is protected by random scalars, ensuring full unlinkability across sessions.

(6) Resistance to Known Session Key Attacks: The session key (*K*) is strongly dependent on ephemeral secrets ($d_{i}^{\prime },d_{j}^{\prime }$) and fresh nonces ($r_{i},r_{j}$). The leakage of a specific session key does not compromise previous or future sessions, satisfying forward/backward secrecy.

(7) Robustness against Physical Attacks: The security root is anchored in the SDL PUF, which is inherently unclonable. Even if storage parameters are compromised, the adversary cannot extract the device-specific fingerprints required to regenerate session keys.

(8) Reduction of Denial-of-Service Attack Impact: The protocol employs lightweight primitives (Hash and XOR) and performs early-stage timestamp checks. This minimizes resource consumption and prevents malicious requests from exhausting the computational capacity of entities.

## 7. Performance Analysis

We benchmark the computational overhead, communication overhead, and security of our proposed protocol against recent state-of-the-art schemes [33,34,37,38,40,44]. While existing designs incorporate PUFs, our solution uniquely integrates the SDL PUF across all network entities (vehicles, TAs, and RSUs) to establish a joint hardware root of trust [49]. Furthermore, by enabling a TA-independent parameter update phase and deploying the CRP obfuscation mechanism for localized storage, our protocol significantly outperforms existing architectures in resisting advanced ML/DL modeling attacks.

### 7.1. Comparison of Security Features

To evaluate the comprehensive security of the proposed protocol, we benchmark it against existing related schemes [61]. In Table 5, “Yes” denotes resistance to a specific attack or support for a feature, while “No” indicates vulnerability or absence. Criteria T1–T11 correspond to the formal proofs (Theorems 1–3 in Section 6, “Formal Security Analysis”) and the heuristic evaluations (items 1–8 in Section 6.2, “Informal Security Analysis”).

As prior literature highlights [12], numerous existing schemes exhibit structural vulnerabilities to modeling and physical attacks due to inherent design flaws. These vulnerabilities primarily stem from the exposure of raw challenge–response pairs (CRPs) over public channels or their storage in plaintext format [22,23,29,30,42], offering only partial resistance via predictable conventional PUFs or weak cryptographic transformations [24,34,37,40,44], or relying exclusively on traditional cryptographic primitives without a hardware root of trust [33,38]. To address these fundamental limitations, our protocol leverages the DRO-Obfuscate mechanism. Table 5 summarizes our protocol’s superior resistance capabilities against these threats compared to the state of the art.

### 7.2. Communication Overhead Comparison

**Figure 4:**
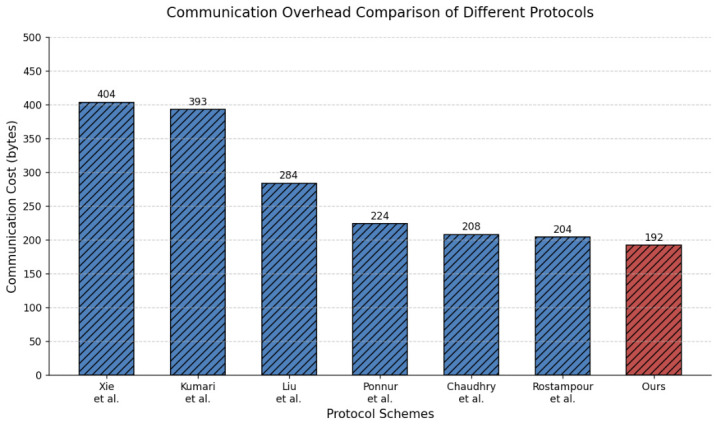
Communication cost comparison with related protocols [33,34,37,38,40,44].

In Figure 4, our protocol’s communication overhead is compared with other relevant protocols. The communication cost calculation is based on public data transmitted between the vehicle and RSU nodes during the authentication and session key agreement phase [13]. The lengths of real identities, pseudo-identities, random numbers, and SHA-1 hash outputs are all assumed to be 160 bits, and timestamps are 32 bits. An ECC point containing coordinates (X,Y) has a size of 160+160=320 bits [62]. In our protocol, ${\mathrm{MSG}}_{1}=\left\{{\mathrm{PID}}_{i},A_{i},D_{i},r_{i},T_{i}\right\}$ has a total data size of 160+160+160+160+32=672 bits, ${\mathrm{MSG}}_{2}=\left\{{\mathrm{PID}}_{i}^{1},\rho_{j},D_{j},r_{j},T_{j}\right\}$ has a total data size of 160+160+160+160+32=672 bits, and ${\mathrm{MSG}}_{3}=\left\{\tau_{i},T_{i}^{*}\right\}$ has a total data size of 160+32=192 bits. The total communication cost is ${\mathrm{MSG}}_{1}+{\mathrm{MSG}}_{2}+{\mathrm{MSG}}_{3}=1536$ bits =192 bytes. Compared to the schemes of Xie et al. [34], Kumari et al. [38], Liu et al. [40], Ponnuru et al. [44], Chaudhry et al. [33], and Rostampour et al. [37], our proposed protocol reduces communication overhead by approximately 52.5%, 51.1%, 32.4%, 14.3%, 7.7%, and 5.9%, respectively, demonstrating superior transmission efficiency.

### 7.3. Computational Overhead Analysis

**Figure 5:**
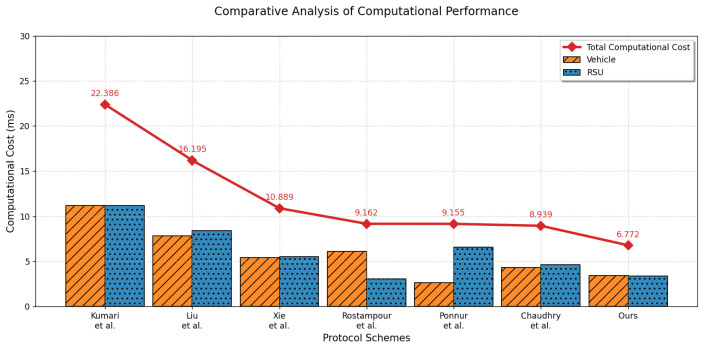
Computational cost comparison with related protocols [33,34,37,38,40,44].

Computational time cost refers to the time the vehicle and RSU consume during identity authentication and key agreement. Since the compared protocols involve hash functions and XOR operations and some include a PUF—a hardware component embedded in participants with negligible time overhead—this section calculates the actual computational costs of the aforementioned protocols using the Python 3.7 programming language. The average execution time is derived after performing 100,000 iterations of each operation. The SHA-1 hash function from Python’s built-in hashlib [63] library is employed for hash computations. For PUF-related operations, the following libraries are used: pypcryptodomex [64], pypuf [65], and python-fuzzy-extractor [66]. Table 6 shows the actual computational costs of the cryptographic primitives used in the protocols. The comparative computational overheads are illustrated in Figure 5 and detailed in Table 7.

### 7.4. Resisting Modeling Attacks

**Figure 6:**
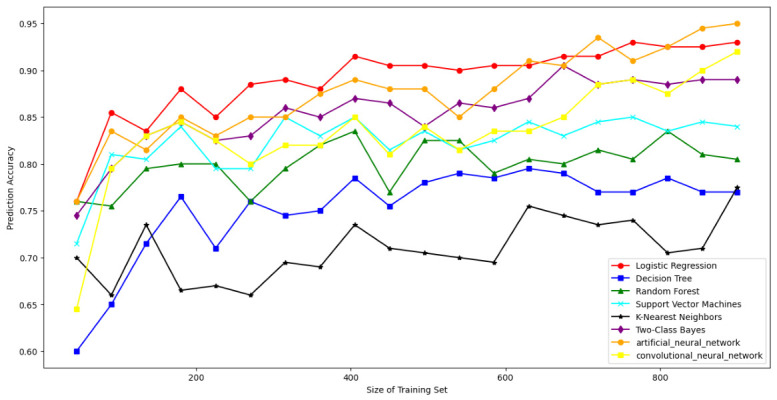
The results of SDL PUF CRP without obfuscation.

**Figure 7:**
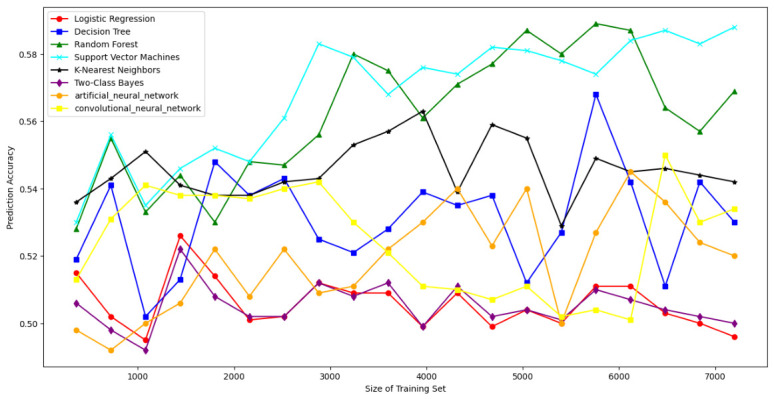
The obfuscation results of the SDL PUF CRP algorithm.

**Figure 8:**
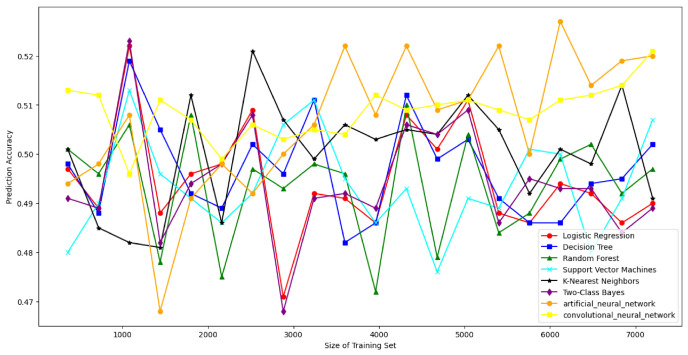
The confusion result of SDL PUF CRP in the agreement.

To rigorously evaluate the robustness of the DRO-Obfuscate mechanism against modeling attacks [67], we deployed a comprehensive multi-dimensional ML/DL attack suite. Instead of limiting the evaluation to basic linear models, our framework encompasses a full spectrum of classifiers: statistical and non-linear mapping models (Logistic Regression [5], Two-Class Bayes [12], SVM [5], Random Forest [12], K-Nearest Neighbors [5], and Decision Trees [5]), as well as deep feature extraction architectures (Artificial Neural Networks (ANNs) [52] and Convolutional Neural Networks (CNNs)) [52]. This ensures full-strength security validation across diverse threat boundaries [68]. As shown in Figure 6, Figure 7 and Figure 8, when raw SDL PUF CRPs are transmitted directly, the prediction accuracy of modeling attacks approaches 97% for 1000 users. However, with our DRO-Obfuscate algorithm applied during the registration phase, the prediction accuracy is strictly bounded to approximately 50–58%. This effectively reduces the attack success rate by roughly 35%. Compared to standard obfuscation methods, our protocol achieves maximum modeling resistance with minimal lightweight overhead.

## 8. Implementation

**Figure 9:**
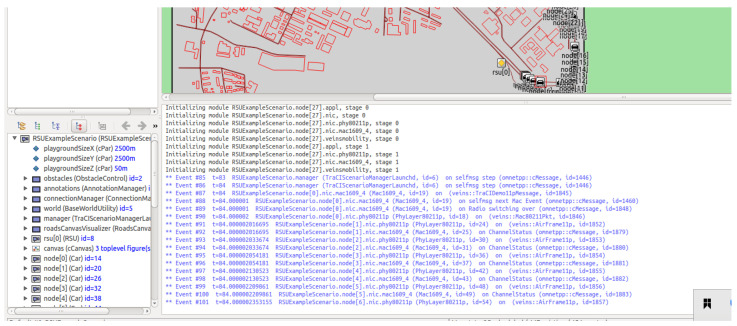
Diagram of the V2X simulation scenario for multiple vehicles. (The red frame indicates the currently running vehicle unit.)

To evaluate the practicality and suitability of the proposed protocol within V2X networks, a comprehensive co-simulation environment was established on an Ubuntu 20.04.6 LTS system equipped with an Intel(R) Core(TM) i5-8300H CPU (2.30 GHz). The experimental framework integrates the OMNeT++ 5.6 simulation platform with the Veins 5.0 framework and SUMO 1.18.0 to facilitate high-fidelity network–traffic coupling. RSUs were deployed at fixed 200-m intervals along a linear trajectory, utilizing IEEE 802.11p wireless interfaces operating on a 5.9 GHz band with a 6 MHz channel bandwidth and 23 dBm transmission power. Vehicle mobility followed a constant 30 m/s model in SUMO, with the network scale varied from 50 to 500 nodes to rigorously assess performance under diverse traffic densities. Each vehicle continuously monitored the proximal RSU via the TraCI interface, triggering the authentication procedure upon RSU handover within a 150-m coverage radius. The primary simulation parameters are detailed in Table 8, and a diagram of the the V2X simulation scenario is shown in Figure 9.

To simulate realistic computational overhead, discrete processing latencies of 49.677 μs (vehicle-side) and 40.637 μs (RSU-side) were incorporated during the exchange of authentication messages. The system addressed potential authentication failures or 800 ms timeouts through an exponential backoff retry mechanism limited to three attempts. Utilizing a log-distance path-loss model with a path-loss exponent of 2.2, the 600-s simulation quantified the impact of varying vehicle conditions on authentication efficiency, end-to-end delay, and throughput.

### 8.1. End-to-End Delay

**Figure 10:**
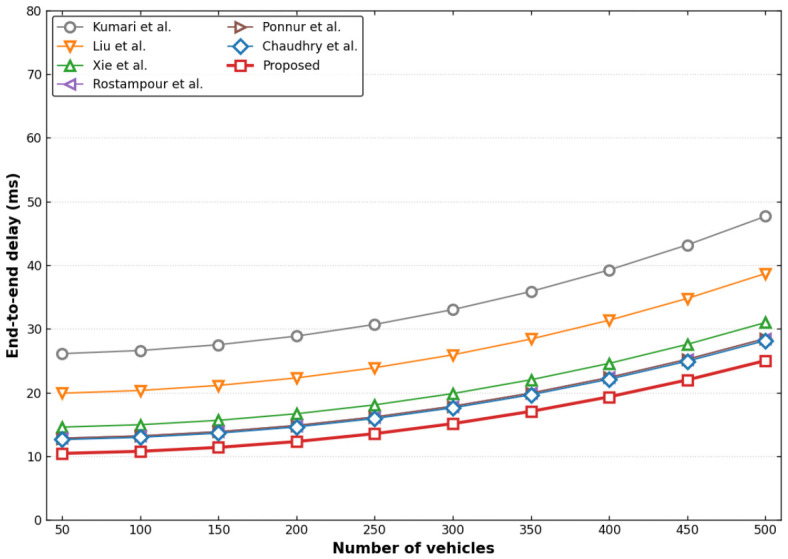
Comparison chart of end-to-end delay for multiple vehicles [33,34,37,38,40,44].

As illustrated in Figure 10, the end-to-end delay [69] of all evaluated protocols exhibits pronounced non-linear growth as vehicle density scales from 50 to 500, reflecting the cumulative latency from Media Access Control (MAC) contention, backoff, and retransmissions in dense V2X environments. However, our proposed protocol consistently maintains the lowest latency profile. Even at peak load (500 vehicles), its delay is constrained to approximately 25 ms, significantly outperforming the schemes of Kumari et al. [38] (48 ms) and Liu et al. [40] (39 ms). This latency resilience stems directly from our streamlined message architecture (192 bytes per authentication cycle) and optimized total computation time (6.772 ms), which collectively suppress the “processing bottleneck-queue overflow” coupling problem under high concurrency.

### 8.2. Emulation Authentication Execution Time

**Figure 11:**
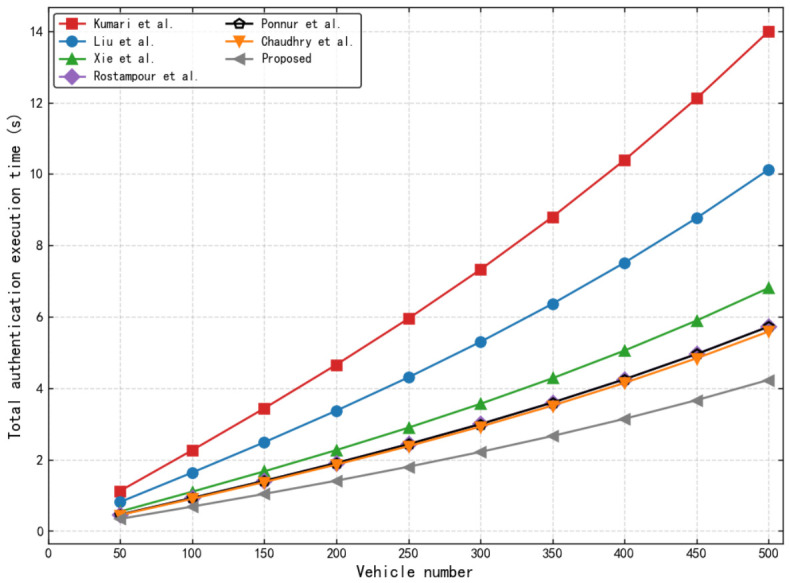
Comparison chart of emulation authentication execution time for multiple vehicles [33,34,37,38,40,44].

As shown in Figure 11, the cumulative authentication execution time exhibits non-linear growth with vehicle density, experiencing a marked acceleration beyond 300 vehicles due to channel contention and backoff mechanisms in high-density V2X environments. Despite this, our proposed protocol maintains superior scalability, demonstrating sustained low-gradient growth suitable for large-scale real-time applications. At a peak load of 500 vehicles, its execution time is restricted to approximately 4.25 s, significantly outperforming the scheme of Kumari et al. [38] (approx. 14 s). This performance advantage is achieved by integrating the SDL PUF, which eliminates the overhead of iterative fuzzy extractors and reduces per-vehicle latency to 3.386 ms. Consequently, the shortened computational loop effectively minimizes MAC-layer queuing and mitigates congestion-induced timeouts.

### 8.3. Throughput

**Figure 12:**
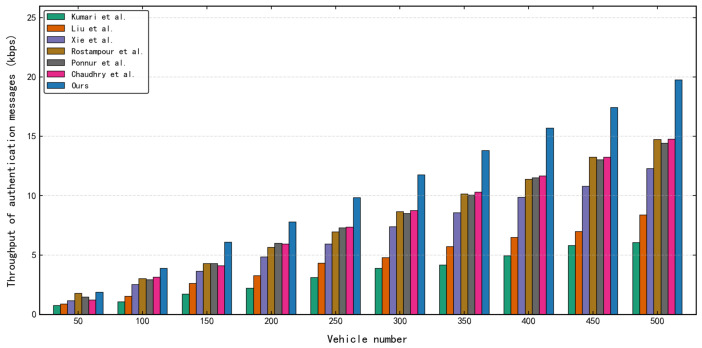
Comparison chart of throughput for multiple vehicles [33,34,37,38,40,44].

As shown in Figure 12, the authentication throughput [69] of all evaluated protocols increases as the vehicle node scale expands from 50 to 500. However, our proposed protocol demonstrates a pronounced performance advantage. At the peak load of 500 vehicles, its throughput approaches 20 kbps, exhibiting significantly greater elasticity compared to the schemes of Kumari et al. [38] and Liu et al. [40]. This superior performance is driven by the minimal data exchange (192 bytes per authentication cycle), which significantly compresses the serialized airtime occupation on the physical channel, and the exceptionally low processing overhead (6.772 ms), which narrows the transaction locking window for individual sessions. Consequently, this architectural efficiency effectively suppresses application-layer bottlenecks and request backlogs under high concurrency, validating its robust suitability for large-scale dynamic network environments.

## 9. Conclusions

This paper proposes a decentralized anonymous V2X authentication protocol based on dynamic PUFs to address the critical security and efficiency challenges in V2X communication. By integrating ECC with lightweight cryptographic primitives, the protocol balances strong security and resource efficiency, making it suitable for resource-constrained on-board environments. The introduction of a dynamic CRP mechanism and the dynamic CRP confusion algorithm for the storage parameters of RSUs and vehicles significantly enhances the resistance to ML- and DL-based modeling attacks, reducing the prediction success rate by 35% compared to independent SDL PUF implementations. Additionally, the protocol ensures user anonymity, session independence, and untraceability through anonymous identifiers and temporary session keys. Formal security verification using the ROR model and AVISPA and simulations in SUMO and Omnetpp confirm the protocol’s resistance to known attacks and its practicality in real-world V2X scenarios. The proposed solution has lower computational and storage overheads than existing schemes, demonstrating its suitability for real-time, large-scale on-board networks.

Future work will focus on integrating the principles of post-quantum cryptography with SDL PUF technology to enhance the protocol’s security further.

## Figures and Tables

**Table 1:** Symbols and their descriptions.

Symbol	Description
${\mathrm{ID}}_{i},\;{\mathrm{ID}}_{j}$	Vehicle and RSU unique identities
${\mathrm{PID}}_{i},\;{\mathrm{PID}}_{i}^{1}$	Vehicle forgery identity
${\mathrm{TID}}_{t}$	TA unique forgery SDL PUF response
${\mathrm{PUF}}_{\mathrm{TA}}$	The SDL PUF of TA
${\mathrm{PUF}}_{V}$	The SDL PUF of the vehicle
${\mathrm{PUF}}_{\mathrm{RSU}}$	The SDL PUF of RSU
$\left(C_{V},R_{V}\right)$	Vehicle PUF challenge–response pair
$\left(C_{\mathrm{RSU}},R_{\mathrm{RSU}}\right)$	RSU PUF challenge–response pair
$\left(C_{\mathrm{TA}},{\mathrm{ID}}_{t}\right)$	TA PUF challenge–response pair
*G*	Cyclic additive group
q,P	Order and generator of *G*
$\left(\alpha_{i},X_{i}\right)$	Vehicle public–private key pair
$\left(\beta_{j},Y_{j}\right)$	RSU public–private key pair
$T_{i},\;T_{j},\;T_{i}^{*}$	Timestamps produced in the agreement
*K*	Vehicle and RSU session key
‖	Concatenation operation
H(·)	Hash function
⊕	XOR operation

**Table 2:** Vehicle registration phase.

Setup Phase	
**Vehicle**	**TA**
Choose ${\mathrm{ID}}_{i}$, $C_{V}$	Verify ${\mathrm{ID}}_{i}$
	Select $\alpha_{i}\in Z_{q}^{*}$, $C_{T}$
	Compute $X_{i}=\alpha_{i}\cdot P$
	Compute ${\mathrm{ID}}_{t}={\mathrm{PUF}}_{\mathrm{TA}}\left(C_{T}\right)$
	$\langle \alpha_{i},X_{i},{\mathrm{ID}}_{t}\rangle :\mathrm{Vehicle}\leftarrow \mathrm{TA}$
Compute $R_{V}={\mathrm{PUF}}_{V}\left(C_{V}\right)$	
Compute $e_{i}=R_{V1}⊕\alpha_{i}$	
Compute $f_{i}=R_{V2}⊕{\mathrm{ID}}_{t}$	
Store $\left\{e_{i},\;C_{V},\;f_{i}\right\}$	
Publish $X_{i}$	

**Table 3:** RSU node registration phase.

Setup Phase	
**RSU**	**TA**
Choose ${\mathrm{ID}}_{j}$, $C_{\mathrm{RSU}}$	Verify ${\mathrm{ID}}_{j}$
	Select $\beta_{j}\in Z_{q}^{*}$, $C_{T}$
	Compute $Y_{j}=\beta_{j}\cdot P$
	Compute ${\mathrm{ID}}_{t}={\mathrm{PUF}}_{\mathrm{TA}}\left(C_{T}\right)$
	$\langle \beta_{j},Y_{j},{\mathrm{ID}}_{t}\rangle :\mathrm{RSU}\leftarrow \mathrm{TA}$
Compute $R_{\mathrm{RSU}}={\mathrm{PUF}}_{\mathrm{RSU}}\left(C_{\mathrm{RSU}}\right)$	
Compute $f_{j}=R_{\mathrm{RSU}1}⊕{\mathrm{ID}}_{t}$	
Compute $e_{j}=R_{\mathrm{RSU}2}⊕\beta_{j}$	
Store $\left\{e_{j},C_{\mathrm{RSU}},f_{j}\right\}$	
Publish $Y_{j}$	

**Table 4:** The process of mutual authentication and key agreement phase.

Vehicle		RSU
Generate $d_{i},\gamma_{i},r_{i}\in Z_{q}^{*}$		
Select challenge $C_{V}$		
Compute $R_{V}={\mathrm{PUF}}_{V}\left(C_{V}\right)$		
Derive $\alpha_{i}=e_{i}⊕R_{V1},\;{\mathrm{ID}}_{t}=f_{i}⊕R_{V2}$		
Compute $d_{i}^{\prime }=H\left(d_{i}∥R_{V}\right),\;D_{i}=d_{i}^{\prime }\cdot P$		
Compute ${\mathrm{PID}}_{i}={\mathrm{ID}}_{i}⊕\gamma_{i}$		
Generate timestamp $T_{i}$		
Compute $A_{i}=H({\mathrm{PID}}_{i}∥{\mathrm{ID}}_{i}∥{\mathrm{ID}}_{j}∥\alpha_{i}Y_{j}∥D_{i}∥T_{i}∥r_{i})$		
Send ${\mathrm{MSG}}_{1}=\left\{{\mathrm{PID}}_{i},A_{i},D_{i},r_{i},T_{i}\right\}$	→	Check $T_{i}$, verify ${\mathrm{ID}}_{j}$
		Generate $d_{j},\gamma_{j},r_{j}\in Z_{q}^{*}$
		Select $C_{\mathrm{RSU}}$
		Compute $R_{\mathrm{RSU}}={\mathrm{PUF}}_{\mathrm{RSU}}\left(C_{\mathrm{RSU}}\right)$
		Derive $\beta_{j}=R_{\mathrm{RSU}1}⊕e_{j},\;{\mathrm{ID}}_{t}=R_{\mathrm{RSU}2}⊕f_{j}$
		Compute $d_{j}^{\prime }=H\left(d_{j}∥R_{\mathrm{RSU}}\right),\;D_{j}=d_{j}^{\prime }\cdot P$
		Compute $K_{DH}=d_{j}^{\prime }\cdot D_{i}$
		Compute $A_{j}=H({\mathrm{PID}}_{i}∥{\mathrm{ID}}_{i}∥{\mathrm{ID}}_{j}∥X_{i}\beta_{j}∥D_{i}∥T_{i}∥r_{i})$
		If $A_{j}\neq A_{i}$, abort
		Generate timestamp $T_{j}$
		Compute ${\mathrm{TID}}_{t}={\mathrm{ID}}_{t}⊕\left(D_{i}\cdot d_{j}^{\prime }\right)$
		Update ${\mathrm{PID}}_{i}^{1}={\mathrm{PID}}_{i}⊕\gamma_{j}$
		Compute $\rho_{j}=H({\mathrm{PID}}_{i}^{1}∥{\mathrm{TID}}_{t}∥A_{j}∥T_{i}∥T_{j}∥r_{j})⊕\left(D_{i}\cdot d_{j}^{\prime }\right)$
		Send ${\mathrm{MSG}}_{2}=\left\{{\mathrm{PID}}_{i}^{1},\rho_{j},D_{j},r_{j},T_{j}\right\}$
	←	
Check $T_{j}$ validity		
Compute $K_{DH}=d_{i}^{\prime }\cdot D_{j}$		
Compute ${\mathrm{TID}}_{t}={\mathrm{ID}}_{t}⊕\left(D_{j}\cdot d_{i}^{\prime }\right)$		
Compute $\rho_{i}=H({\mathrm{PID}}_{i}^{1}∥{\mathrm{TID}}_{t}∥A_{i}∥T_{i}∥T_{j}∥r_{j})⊕\left(D_{j}\cdot d_{i}^{\prime }\right)$		
If $\rho_{i}\neq \rho_{j}$, abort		
Generate $T_{i}^{*}$		
Compute session key $K=H(K_{DH}∥{\mathrm{PID}}_{i}∥{\mathrm{PID}}_{i}^{1}∥T_{i}∥T_{j}∥r_{i}∥r_{j})$		
Compute $\tau_{i}=H\left(K∥T_{i}^{*}\right)$		
Select $C_{V}^{\prime }$, compute $R_{V}^{\prime }={\mathrm{PUF}}_{V}\left(C_{V}^{\prime }\right)$, update $f_{i},e_{i}$		
Send ${\mathrm{MSG}}_{3}=\left\{\tau_{i},T_{i}^{*}\right\}$	→	Check $T_{i}^{*}$ validity
		Compute $K=H(K_{DH}∥{\mathrm{PID}}_{i}∥{\mathrm{PID}}_{i}^{1}∥T_{i}∥T_{j}∥r_{i}∥r_{j})$
		Compute $\tau_{j}=H\left(K∥T_{i}^{*}\right)$
		If $\tau_{i}\neq \tau_{j}$, abort
		Select $C_{\mathrm{RSU}}^{\prime }$, compute $R_{\mathrm{RSU}}^{\prime }$, update $f_{j},e_{j}$
**Mutual authentication and key agreement completed**		

**Table 5:** Comparison of security features across relevant protocols.

Scheme/Feature	T1	T2	T3	T4	T5	T6	T7	T8	T9	T10	T11	ML and DL
Guajardo et al. [22]	Yes	Yes	Yes	Yes	No	Yes	Yes	Yes	No	Yes	Yes	Vulnerable
Sadeghi et al. [23]	Yes	Yes	Yes	Yes	No	Yes	Yes	No	Yes	Yes	Yes	Vulnerable
Van Herrewege et al. [24]	Yes	Yes	Yes	Yes	No	No	Yes	Yes	No	Yes	Yes	Partial
Yanambaka et al. [29]	Yes	Yes	Yes	No	Yes	Yes	Yes	No	Yes	Yes	Yes	Vulnerable
Long et al. [30]	Yes	Yes	No	No	Yes	Yes	No	Yes	Yes	Yes	No	Vulnerable
Men et al. [42]	Yes	Yes	No	Yes	Yes	Yes	No	No	Yes	No	Yes	Vulnerable
Xie et al. [34]	Yes	Yes	Yes	Yes	No	Yes	Yes	Yes	Yes	Yes	No	Basic
Liu et al. [40]	Yes	Yes	Yes	Yes	Yes	Yes	Yes	No	Yes	Yes	No	Partial
Chaudhry et al. [33]	Yes	Yes	No	Yes	No	Yes	No	Yes	Yes	Yes	Yes	-
Rostampour et al. [37]	Yes	Yes	Yes	Yes	Yes	Yes	Yes	Yes	Yes	No	Yes	Basic
Kumari et al. [38]	Yes	Yes	Yes	Yes	Yes	Yes	Yes	Yes	Yes	No	Yes	-
Ponnuru et al. [44]	Yes	Yes	Yes	Yes	Yes	Yes	Yes	Yes	Yes	Yes	Yes	Partial
ours	Yes	Yes	Yes	Yes	Yes	Yes	Yes	Yes	Yes	Yes	Yes	Strong

**Table 6:** Configuration of the emulated device.

Notation	Description	Run Time (ms)
$t_{h}$	Hash operation	0.001
$t_{ecm}$	Scale multiplication on ECC	1.017
$t_{ep}$	Modular exponentiation	1.017
$t_{eca}$	Point addition on ECC	0.051
$t_{aes}$	AES-256 encryption/decryption	0.265
$t_{puf}$	PUF operation	0.324
$t_{xor}$	XOR operation	0.001
$t_{ge}$	Fuzzy generation operation	0.637
$t_{fe}$	Fuzzy extraction operation	0.229

**Table 7:** Computational cost comparison with relevant protocols.

Scheme	Vehicle (ms)	RSU (ms)	Total (ms)
Liu et al. [40]	$t_{\mathrm{puf}}+9t_{h}+2t_{\mathrm{eca}}+7t_{\mathrm{ecm}}+t_{\mathrm{xor}}+t_{\mathrm{fe}}\approx 7.784$	$t_{\mathrm{puf}}+6t_{h}+2t_{\mathrm{eca}}+7t_{\mathrm{ecm}}+t_{\mathrm{xor}}+t_{\mathrm{fe}}\approx 8.411$	16.195
Xie et al. [34]	$5t_{\mathrm{ecm}}+t_{\mathrm{eca}}+5t_{h}+t_{\mathrm{fe}}+2t_{\mathrm{xor}}\approx 5.372$	$5t_{\mathrm{ecm}}+2t_{\mathrm{eca}}+4t_{h}+t_{\mathrm{puf}}+2t_{\mathrm{xor}}\approx 5.517$	10.889
Chaudhry et al. [33]	$3t_{\mathrm{ecm}}+5t_{\mathrm{aes}}+4t_{h}\approx 4.335$	$4t_{\mathrm{ecm}}+2t_{\mathrm{aes}}+6t_{h}\approx 4.604$	8.939
Rostampour et al. [37]	$6t_{\mathrm{ecm}}+5t_{h}\approx 6.107$	$3t_{\mathrm{ecm}}+4t_{h}\approx 3.055$	9.162
Kumari et al. [38]	$5t_{\mathrm{ep}}+6t_{\mathrm{ecm}}+6t_{h}\approx 11.193$	$5t_{\mathrm{ep}}+6t_{\mathrm{ecm}}+6t_{h}\approx 11.193$	22.386
Ponnuru et al. [44]	$2t_{\mathrm{ecm}}+t_{\mathrm{puf}}+6t_{h}+t_{\mathrm{xor}}+t_{\mathrm{fe}}\approx 2.594$	$5t_{\mathrm{ecm}}+t_{\mathrm{puf}}+6t_{h}+t_{\mathrm{xor}}+5t_{\mathrm{fe}}\approx 6.561$	9.155
Ours	$t_{\mathrm{puf}}+7t_{\mathrm{xor}}+3t_{\mathrm{ecm}}+4t_{h}\approx 3.386$	$t_{\mathrm{puf}}+7t_{\mathrm{xor}}+3t_{\mathrm{ecm}}+4t_{h}\approx 3.386$	6.772

**Table 8:** Simulation parameters.

Parameter	Value
Wireless communication standard	IEEE 802.11p
Operating frequency	5.9 GHz
Number of RSUs	10
Number of vehicles	50, 100, …, 500
RSU spacing	200 m
RSU coverage range	150 m
Propagation loss model	Log-distance (exponent 2.2)
Channel bandwidth	6 MHz
Vehicle mobility model	Constant velocity mobility model
Vehicle speed	30 m/s
Transmitted power	23 dBm
Vehicle processing delay	49.677 μs
RSU processing delay	40.637 μs
Authentication timeout per attempt	800 ms
Maximum number of re-authentications per vehicle	3
Simulation time	600 s
Simulation platform	OMNeT++ 5.6 + Veins 5.0 + SUMO 1.18.0
Operating system	Ubuntu 20.04.6 LTS

## Data Availability

No new data were created or analyzed in this study.

## Abbreviations

The following abbreviations are used in this manuscript:

VANET	Vehicular Ad Hoc Network
V2X	Vehicle to Everything
VAKA	Vehicular Authentication and Key Agreement
ECC	Elliptic Curve Cryptography
ROR	Real Or Random
SDL PUF	Self-Adaption Deviation Locking PUF
MITM	Man In The Middle
DoS	Denial Of Service
